# Strangulated Caecum, Appendix, and Terminal Ileum in Paraumbilical Hernia: An Unusual Presentation in the Elderly

**DOI:** 10.7759/cureus.33180

**Published:** 2022-12-31

**Authors:** Reda H Mithany, Ahmed Nazari

**Affiliations:** 1 Laparoscopic Colorectal Surgery, Northampton General Hospital, Northampton, GBR; 2 Colorectal Surgery, Northampton General Hospital, Northampton, GBR

**Keywords:** computed tomography, mobile coecum, malrotation, strangulation, intestinal obstruction, hernia

## Abstract

A common surgical condition caused by inadequate closure of the embryonic umbilical defect is an umbilical hernia. The paraumbilical hernia sac usually contains the omentum and bowel loop, although the caecum is infrequently present. The rare incidence of the caecum being present in the paraumbilical hernial sac is described in a few case reports in the literature. In this article, we'd like to discuss a case of a 71-year-old woman who presented with a paraumbilical hernia with strangulated caecum, appendix, and terminal ileum. Right hemicolectomy, end ileostomy, and transverse colon mucus fistula have been performed with uneventful recovery achieved.

## Introduction

A congenital or acquired condition called paraumbilical hernia is brought on by an inadequate closure of the embryonic umbilical defect. Paraumbilical hernia represents 10% of abdominal wall hernias; the occurrence being 90% in Africans and 10% in Caucasians. Although strangulation is not common, the majority of paraumbilical hernias are reducible and manifest as a lump [1,2]. The sac's most frequent contents are the omentum and bowel loop, although the caecum is only very infrequently present, and surgery is still the only curative option [3]. We discuss a case of a 71-year-old woman who presented with a paraumbilical hernia with strangulated caecum, appendix, and terminal ileum. Right hemicolectomy, end ileostomy, and transverse colon mucus fistula were performed with uneventful recovery achieved.

## Case presentation

A 71-year-old female presented to Accident and Emergency feeling increasingly unwell for a week with vomiting, abdominal pain, and anuria for the past four days. She required fluid resuscitation on admission and was noted to have fast atrial fibrillation secondary to sepsis. The patient had a history of Henoch-Schönlein purpura, hypertension, chronic venous leg ulcers, and chronic kidney disease stage two. 

**Figure 1:**
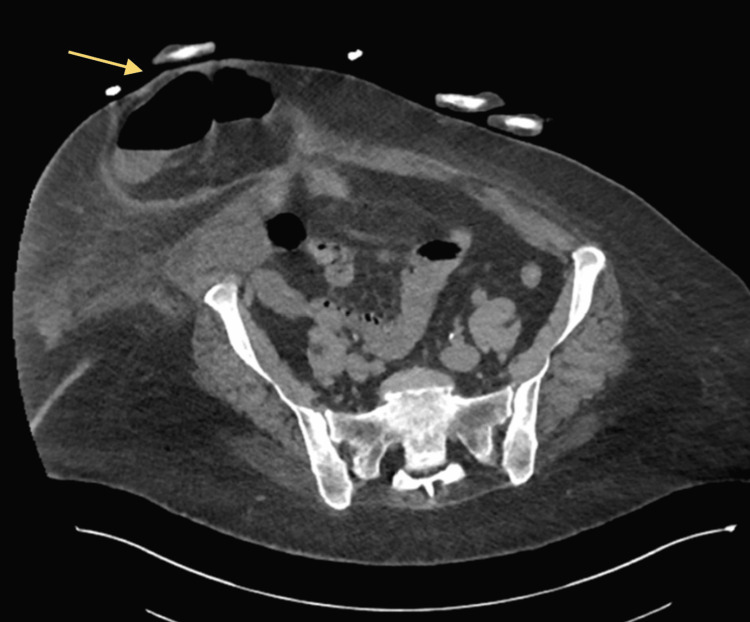
CT abdomen and pelvis: large paraumbilical hernia secondary with a neck of 3cm.

On examination, the patient was noted to have a paraumbilical mass and the abdomen was distended and tender all over with gargling bowel sounds on auscultation. Blood tests showed raised inflammatory markers with white blood cells of 25.5 x 10^9 g/L. CT abdomen and pelvis with intravenous contrast showed a large paraumbilical hernia approximately 12cm in size containing intestinal contents (Figure 1).

**Figure 2:**
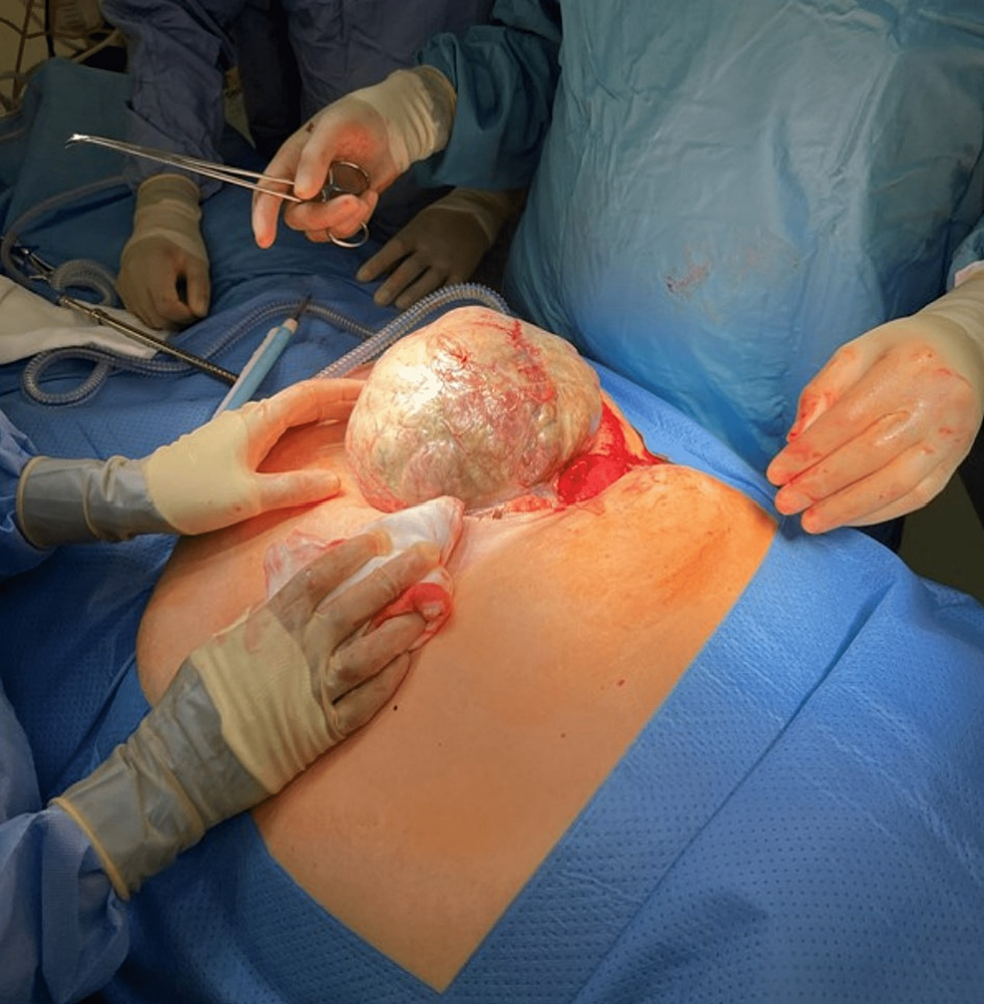
Hernial sac during emergency laparotomy.

The patient underwent an emergency laparotomy that revealed gangrenous caecum, appendix, and terminal ileum in the hernial sac with an offensive smell (Figure 2).

The decision was made to perform a right hemicolectomy. An ileostomy and a transverse colon mucous fistula were fashioned on the right side. Post-operatively, the patient recovered gradually over five days in the intensive care unit, then stepped down to level one care. 

## Discussion

It remains unclear how the caecum can move freely to the midline and present inside the sac of a paraumbilical hernia. The possibility of an anatomical variation and aberrant cecum mobility, also known as mobile cecum syndrome, in 10% of the population has been reported [4].

Clinical examination is crucial in these cases (irreducible, incarcerated, and strangulated hernias), and it's arguable that CT is still the optimum investigation of choice in uncertain situations, particularly when there is a suspicion of malignancy in the elderly. However, surgery is the only effective treatment for strangulated hernia, and a careful inspection of the contents of the sac before reduction and/or resection-anastomosis [5].

Embryologically, the posterior peritoneum typically attaches to the cecum and the ascending colon retroperitoneally [6]. The right ascending colon and caecum may become loose and excessively mobile if the right colonic mesentery fails to fuse to the lateral peritoneum. Long floppy mesentery attached to the retroperitoneum by a limited base of origin is another cause of the mobile caecum [7].

A rare but serious occurrence is caecal wall necrosis related to incarcerated Richter's femoral hernia. This has been reported in one case presentation earlier, which, in comparison to our case, is similar in terms of unusual caecum mobility [8].

A very similar case has been published recently that describes a large, incarcerated umbilical hernia in an obese, 80-year-old patient. Over a period of more than 15 years, the hernia persisted and grew bigger and bigger. The pain, which lasted for almost two hours before the patient arrived at the hospital, was the only symptom. Gangrenous ascending and transverse colon was discovered after an emergency laparotomy. Without hernia repair, a subtotal colectomy with the formation of a terminal ileostomy was performed. The patient was discharged on the fifth postoperative day after a full recovery [9].

## Conclusions

it is rare that a paraumbilical hernia contains caecum and appendix. As demonstrated in our case, the condition is not necessarily fatal; high index of suspicion, proper clinical evaluation, and urgent management are crucial. Our case suggests that right hemicolectomy with ileostomy and mucus fistula are the options of choice to limit contamination and enhance early recovery. Strangulated paraumbilical hernia can be successfully managed with early surgical intervention; hence, it is essential to be aware of this condition and its misleading clinical presentation.
